# Extended methods for influence maximization in dynamic networks

**DOI:** 10.1186/s40649-018-0056-8

**Published:** 2018-10-01

**Authors:** Tsuyoshi Murata, Hokuto Koga

**Affiliations:** Department of Computer Science, School of Computing, Tokyo Institute of Technology, W8-59 2-12-1 Ookayama, Meguro, Tokyo, 152-8552 Japan

**Keywords:** Influence maximization problem, Dynamic networks, SI model

## Abstract

**Background:**

The process of rumor spreading among people can be represented as information diffusion in social network. The scale of rumor spread changes greatly depending on starting nodes. If we can select nodes that contribute to large-scale diffusion, the nodes are expected to be important for viral marketing. Given a network and the size of the starting nodes, the problem of selecting nodes for maximizing information diffusion is called influence maximization problem.

**Methods:**

We propose three new approximation methods (Dynamic Degree Discount, Dynamic CI, and Dynamic RIS) for influence maximization problem in dynamic networks. These methods are the extensions of previous methods for static networks to dynamic networks.

**Results:**

When compared with the previous methods, MC Greedy and Osawa, our proposed methods were found better than the previous methods: Although the performance of MC greedy was better than the three methods, it was computationally expensive and intractable for large-scale networks. The computational time of our proposed methods was more than 10 times faster than MC greedy, so they can be computed in realistic time even for large-scale dynamic networks. When compared with Osawa, the performances of these three methods were almost the same as Osawa, but they were approximately 7.8 times faster than Osawa.

**Conclusions:**

Based on these facts, the proposed methods are suitable for influence maximization in dynamic networks. Finding the strategies of choosing a suitable method for a given dynamic network is practically important. It is a challenging open question and is left for our future work. The problem of adjusting the parameters for Dynamic CI and Dynamic RIS is also left for our future work.

## Background

Diffusion of rumors (or information) can be represented as information propagation in a social network where its nodes are people and its edges are contacts among the people. The scale of information propagation depends on where to start the propagation. In order to propagate as much as possible, starting nodes should be carefully selected. Selecting starting nodes for large-scale information propagation is important as one of the methods for viral marketing.

From given network, selecting such starting nodes for large-scale information propagation was formalized as “influence maximization problem” by Kempe et al. [1]. The original formalization is for static networks. However, nodes and edges can be newly added or deleted in many real social networks. Therefore, influence maximization problem in dynamic networks should be considered. Habiba et al. defined the problem for dynamic networks [2]. Since the problem was proved to be NP-Hard, computing the best solution in realistic time is computationally intractable. Therefore, many approximation methods based on Monte-Carlo simulation and heuristic methods have been proposed. Methods based on Monte-Carlo simulation are accurate but computationally expensive. On the other hand, heuristic methods are fast but they are less accurate.

In order to find better solutions for the information maximization problem, we propose three new methods for dynamic networks as the extension of the methods for static networks. Dynamic Degree Discount is a heuristic method based on node degree. Dynamic CI is a method based on a node’s degree and the degrees of reachable nodes from the node within specific time. Dynamic RIS uses many similar networks generated by random edge removal. We compare the proposed methods with previous methods. The number of propagated nodes based on our method is about 1.5 times of that of previous methods. And computational time of our method is about 7.8 times faster than previous methods.

The authors discuss the extended methods for influence maximization in dynamic networks [3]. In addition to the contents in [3], this paper includes detailed explanation of background knowledge, discussions of the effect of different values of parameters in the proposed methods, and detailed analysis of the advantages and disadvantages of the proposed methods.

The structure of this paper is as follows. “Related work” section shows related work. “Proposed methods” section presents proposed methods (Dynamic Degree Discount, Dynamic CI and Dynamic RIS), “Experiments” section explains our experiments, and “Experimental results” section shows the experimental results. “Discussion” section shows discussions about the experimental results, and “Conclusion” section concludes the paper.

## Related work

### Model of information propagation

We use SI model as the model of information propagation on networks. In SI model, each node in networks is either in state S (susceptible) or in state I (infected). Nodes in state S do not know the information and those in state I know the information. At the beginning of information propagation (at time $t=1$), a set of nodes in state I is fixed as the seed nodes. For all edges (*t*, *u*, *v*) at time $t = 1, 2, \ldots , T$, the following operations are performed. If node *u* is in state I and node *v* in state S, information is propagated from *u* to *v* with probability $\lambda$, which means the state of *v* is changed from S to I at time $t+1$. Probability $\lambda$ is the parameter of susceptibility, and it controls the percentage of information propagation. At time $t=T+1$, information propagation is terminated.

Based on the above notations, we can formulate influence maximization problem as follows. We define $\sigma (S)$ as the expected number of nodes of state I at time $T+1$ when information propagation started at time 1 from seed nodes *S* of state I based on SI model. (Please keep in mind that *S* in $\sigma (S)$ is a set of seed nodes, and *S* in SI model is susceptible state.) Influence maximization problem in a dynamic network is to search for a set of seed nodes *S* of size *k* that maximizes $\sigma (S)$ when a dynamic network *G*, duration of the network *T*, susceptibility of SI model $\lambda,$ and the size of seed nodes *k* are given.

### Problems related to influence maximization in dynamic networks

There are some problems related to influence maximization in dynamic networks. Instead of giving item (or information) to seed nodes for free, revenue maximization [4] is the problems of finding seed customers (nodes) and offering discounts to them in order to increase total revenue. Although the problem is important in the field of marketing, it is more complicated than influence maximization problem since seed nodes are not treated as equal, and the amount of discount for each node may not be equal. The number of possible parameters increases greatly especially in the case of dynamic networks. Although revenue maximization is one of the important research directions, it is different from influence maximization problem.

Opinion formation [5–7] is another problem related to influence maximization problem. Each agent (node) has an opinion which might be a continuous or a discrete quantity. The underlying network represents the society where the agents have interactions. Each agent has an opinion in the society that is influenced by the society. Analyzing the increase and decrease of each opinion is important for modeling the dynamics of opinion formation and for opinion polarization [8].

It is often pointed out that the properties of dynamic networks are quite different from those in static networks. Braha and Bar-Yam [9, 10] pointed out the overlap of the centrality in dynamic networks and that in the aggregated (static) network is very small. Hill and Braha [11] propose dynamic preferential attachment mechanism that reproduce dynamic centrality phenomena. Holme presents good surveys on dynamic networks [12, 13].

### Influence maximization methods for static networks

Jalili presents a survey on spreading dynamics of rumor and disease based on centrality [14]. There are roughly three approaches for influence maximization problem in static networks. The first is Monte-Carlo simulation methods, the second is heuristic-based methods, and the third is the methods to generate a large number of networks with random edge removal and select seed nodes based on the generated networks.

Monte-Carlo simulation method is proposed by Kempe et al. [1]. $\sigma (S)$ is estimated by repeating Monte-Carlo simulation in Kepme’s method. When *S* is given as a set of seed nodes, simulations of information propagation are repeated *R* times and the average number of infected nodes is defined as $\sigma (S)$. Next, the node *v* which maximizes the difference $\sigma (S \cup \{ v \}) - \sigma (S)$ is added to seed nodes greedily based on the estimated $\sigma (S)$. This operation is repeated until $|S|=k$.

Since $\sigma (\cdot )$ is a monotonic and submodular function, when we denote strict solution of seed nodes as $S^{*}$, the seed nodes obtained by the above greedy algorithm ${S_{\text{greedy}}}$ are proved to satisfy $\sigma ({S_{\text{greedy}}}) \ge (1-1/e) \sigma (S^{*})$ [1]. Because of this property, qualities of the solutions by Kempe’s method are good. However, more and more repetition of Monte-Carlo simulation is needed in order to estimate $\sigma (S)$ accurately. Since the computational cost for finding seed nodes with this method is high, it is not possible to find seed nodes in realistic time for large-scale networks.

Heuristic methods are proposed in order to search for seed nodes at high speed. Chen et al. [15] proposes PMIA to find seed nodes focusing on the paths with high information propagation ratio. Jiang et al. [16] proposed SAEDV which searches for seed nodes by annealing method to obtain $\sigma ( \cdot )$ from adjacent nodes in seed nodes. Chen et al. [17] proposed Degree Discount based on node degree where the nodes adjacent to already selected node are given penalty. This is because when node *v* is selected as one of seed nodes and *u* is its neighbor, it is highly likely that *v* propagates information to *u*, so selecting nodes other than *u* as seed nodes is better for information diffusion.

Algorithm of Degree Discount is shown as follows. $t_i$ in the algorithm shows the penalty of node *i*. $\mathrm {dd}_i$ is the degree of node *i* after giving penalty. $\mathrm {dd}_i$ is smaller when the value of $t_i$ is bigger. 
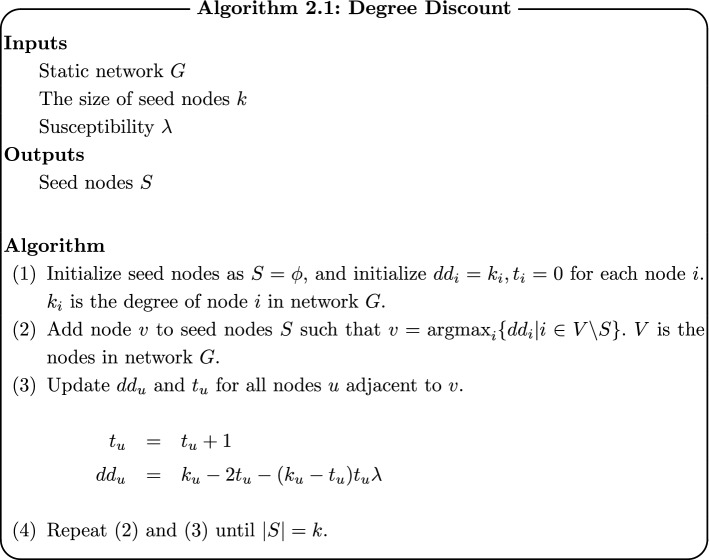


Morone et al. [18] proposed a method for finding seed nodes considering the degrees of distant nodes. The method calculates the following $\mathrm{CI}_l (v)$ for each node and selects seed nodes based on the values:

$$\begin{aligned} \mathrm{CI}_l (v) = (k_v - 1) \sum _{u \in \partial \mathrm {Ball} (v, l)} (k_u - 1). \end{aligned}$$

**Fig. 1:**
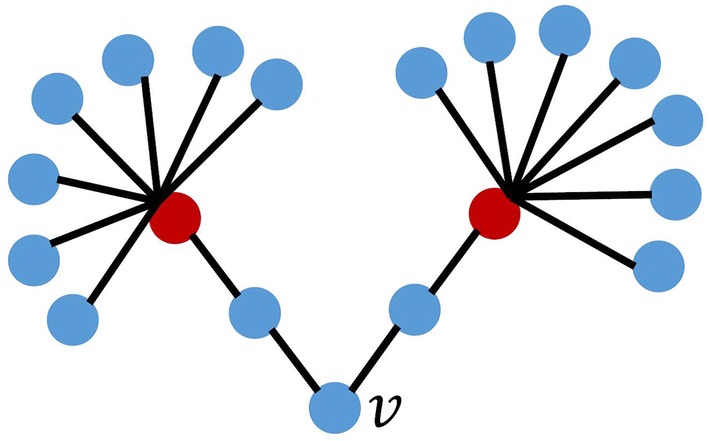
Example for explaining $\mathrm{CI}_l (v)$. When $l=2$, $\mathrm{CI}_{l}(v) = 14$

$\partial \mathrm {Ball} (v, l)$ in the above formula represents nodes where the distance from node *v* is *l*. The example of $\mathrm{CI}_l (v)$ is explained in Fig. 1. $\partial \mathrm {Ball} (v, 2)$ when $l=2$ are two red nodes with distance 2 from node *v* and the degrees of both nodes are 8. Therefore, $\mathrm{CI}_2 (v)=(2-1) \times \{ (8-1)+(8-1) \}=14.$

The degree of node *v* itself is low in the network in Fig. 1, but the node *v* is effective for information propagation because it is connected with some high degree nodes with distance two. This method thus selects seed nodes with wider propagation compared with the cases when seed nodes are selected based on the degree of node *v* only.

These heuristic methods compute seed nodes faster than the methods based on Monte-Carlo simulation. However, it is experimentally confirmed that the scale of propagation of the methods depends on network structures and parameters.

Ohsaka et al. [19] proposed a method to generate many networks with random edge removal in order to solve this problem. Ohsaka’s method is based on “coin flip” mentioned in Kempe’s paper [1]. Distribution of nodes where information is propagated from seed nodes *S* in static network *G* is set as $D_G (S)$. And distribution of nodes where information is propagated from seed nodes *S* on network where edges are removed at constant ratio from the network *G* is set as $D^{\prime }_G (S)$. “Coin flip” means that $D_G (S)$ equals to $D^{\prime }_G (S)$ in this situation, and that $\sigma (\cdot )$ can be estimated by generating many networks with edges removed at constant ratio, not by repeating Monte-Carlo simulation. Ohsaka’s method estimates $\sigma (\cdot )$ by acquiring Strongly Connected Component (SCC) in each network generated by *RR* numbers of networks with edges removed at constant ratio. SCC is a subgraph where each node in the subgraph can be reachable to and from any other nodes.

Borgs et al. [20] and Tang et al. [21] also propose methods similar to Ohsaka’s method. The difference from Ohsaka’s method is $\sigma (\cdot )$, which is not estimated directly from generated networks. Reachable nodes from randomly selected node *v* are computed, and then seed nodes are selected based on the nodes. More specifically, the algorithm is as follows. 
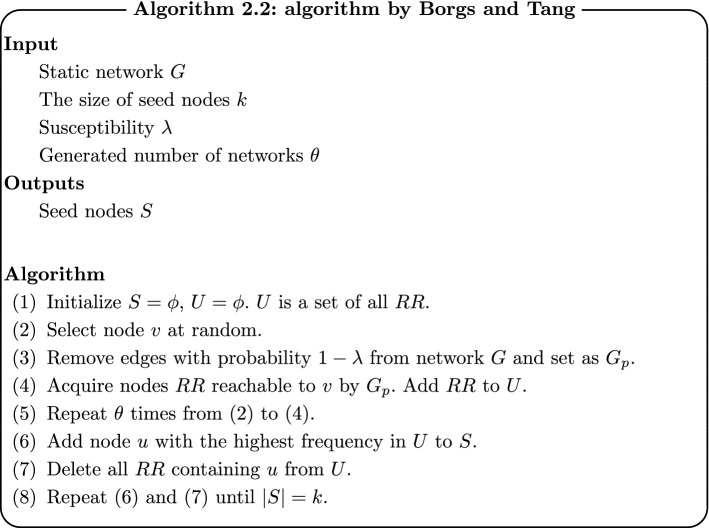


There are other approaches for influence maximization problem in different problem settings. Chen et al. [22] proposed a method to solve the problem with time limit. Feng et al. [23] solves the influence maximization problem in a situation where freshness of the information degrades as it spreads. Mihara et al. [24] proposed a method to influence maximization problem where the whole network structure is unknown.

### Degrees in dynamic networks

Notations of edges and paths in dynamic networks are the same as the ones in ref. [25]. (*t*, *u*, *v*) represents an edge from node *u* to *v* at time *t*. A path from node $v_1$ to $v_k$ of length $k-1$ is represented as $(t_1,v_1,v_2),(t_2,v_2,v_3), \ldots ,(t_{k-1},v_{k-1},v_k)$, where $t_1<t_2<\cdots <t_{k-1}$ and $\forall i,j(i\ne j),v_i \ne v_j$. Duration of time from the start to the end of a path $t_{k-1}-t_1$ is the length of time of the path, and the smallest one is the minimum length of time.

**Fig. 2:**
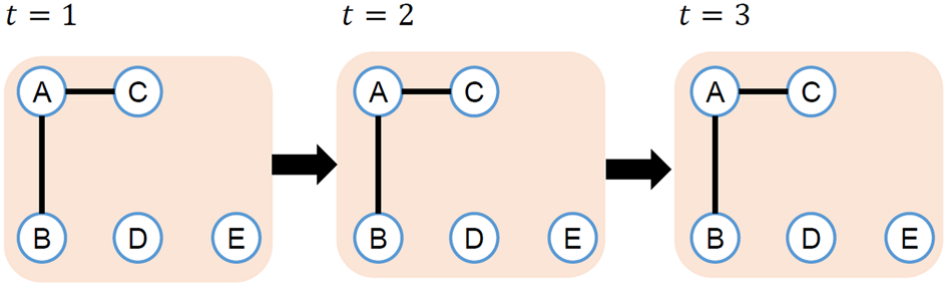
Example of low degree nodes in a dynamic network

**Fig. 3:**
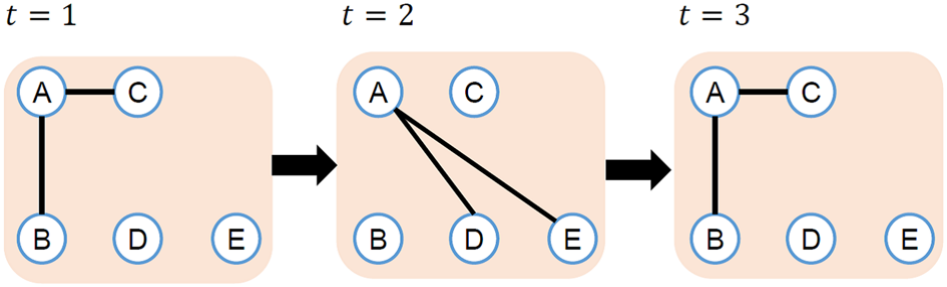
Example of high degree nodes in a dynamic network

Habita et al. [26] define degrees in dynamic network using symmetric difference of past connections and future connections. However, diffusion in dynamic networks is from past to future only, and it is not bidirectional. We therefore define degree $D_T(v)$ of node *v* in dynamic network as follows:

$$\begin{aligned} D_T(v) = \sum _{1<t\le T} \frac{|N (v, t-1) \backslash N (v, t)|}{|N (v, t-1) \cup N(v, t)|} |N(v, t)|, \end{aligned}$$

where *N*(*v*, *t*) is a collection of nodes adjacent to node *v* at time *t*. Figures 2 and 3 illustrate the examples of degrees on dynamic networks. In Fig. 2, adjacent nodes of node *A* do not change during the period. The difference of adjacent nodes $N(\mathrm{A}, 1) \backslash N(\mathrm{A}, 2)$ and $N(\mathrm{A}, 2) \backslash N(\mathrm{A}, 3)$ is empty. Therefore, the degree of node *A* in Fig. 2 is calculated as follows.

$$\begin{aligned} D_3(\mathrm{A})= & {} \frac{|N(\mathrm{A},1) \backslash N(\mathrm{A},2)|}{|N(\mathrm{A},1) \cup N(\mathrm{A},2)|} |N(A,2)|+ \frac{|N(\mathrm{A},2) \backslash N(\mathrm{A},3)|}{|N(\mathrm{A},2) \cup N(\mathrm{A},3)|} |N(A,3)| \\= & {} \frac{0}{2} \times 2 + \frac{0}{2} \times 2 = 0 \end{aligned}$$

On the other hand, in Fig. 3, nodes adjacent to node *A* change over time. So the degree of node *A* is bigger than that in Fig. 2.

$$\begin{aligned} D_3(\mathrm{A})= & {} \frac{|N(\mathrm{A},1) \backslash N(\mathrm{A},2)|}{|N(\mathrm{A},1) \cup N(\mathrm{A},2)|} |N(A,2)|+ \frac{|N(\mathrm{A},2) \backslash N(\mathrm{A},3)|}{|N(\mathrm{A},2) \cup N(\mathrm{A},3)|} |N(A,3)| \\= & {} \frac{2}{4} * 2 + \frac{2}{4} * 2 = 2 \end{aligned}$$

In Figs. 2 and 3, the number of adjacent nodes of node *A* is the same every time, so the average degree of node *A* is the same in Figs. 2 and 3. On the other hand, if we employ $D_T(v)$ as the definition of node degree, $D_3(A)=0$ in Fig. 2, and $D_3(A)=2$ in Fig. 3. $D_T(v)$ captures the number of newly adjacent nodes, and this is important for influence maximization problem. We therefore employ $D_T(v)$ as the definition of node degree in dynamic networks.

### Influence maximization methods for dynamic networks

There are two approaches for influence maximization problem in dynamic networks: methods based on Monte-Carlo simulation and heuristic-based methods. The former method is proposed by Habiba and Berger-Wolf [2]. The method estimates the scale of propagation $\sigma (\cdot )$ by repeating Monte-Carlo simulation just the same as in static networks. Since $\sigma (\cdot )$ is monotonic and deteriorated modular also in dynamic networks, this method achieves large-scale propagation. However, the computational cost of this method is high as in static networks. Osawa and Murata [25] proposed a heuristic method for calculating $\sigma (\cdot )$ at high speed. His algorithm for computing $\sigma (S)$ for seed nodes *S* is shown as follows. 
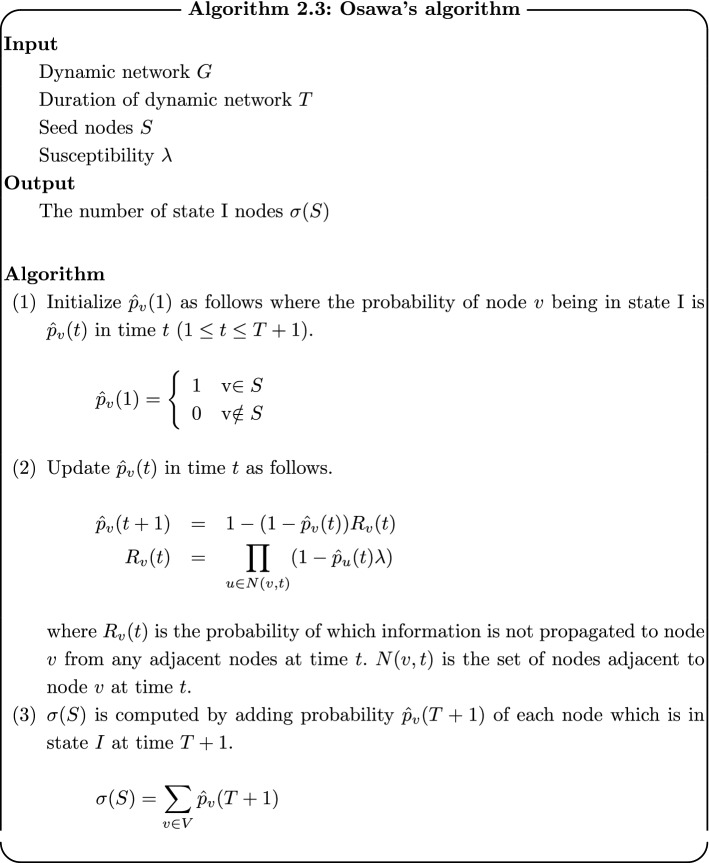


After $\sigma (S)$ is computed, seed nodes are obtained by greedy algorithm as in the method by Monte-Carlo simulation. Osawa’s method finds seed nodes in realistic computational time. However, the quality of its solution depends on given networks because $\sigma (\cdot )$ is calculated approximately, and it is worse compared with the solutions by Monte-Carlo simulation.

## Proposed methods

We propose new methods for influence maximization problem in dynamic networks in this section. We propose three new methods (Dynamic Degree Discount, Dynamic CI, and Dynamic RIS) which are the extensions of static network methods to dynamic network methods. We use the following notations: *G*: dynamic network, *T*: duration of the dynamic network, *k*: the size of seed nodes, $\lambda$: susceptibility, $\theta$: the number of generated networks, and *S*: seed nodes.

### Dynamic Degree Discount

Dynamic Degree Discount is the extension of Degree Discount by Chen et al. [17] to dynamic networks. In Dynamic Degree Discount, definitions of degrees and adjacent nodes in the algorithm of Degree Discount are modified for dynamic networks. Algorithms 3.1 shows the algorithm of Dynamic Degree Discount. Underlines show the parts modified from original Degree Discount. 
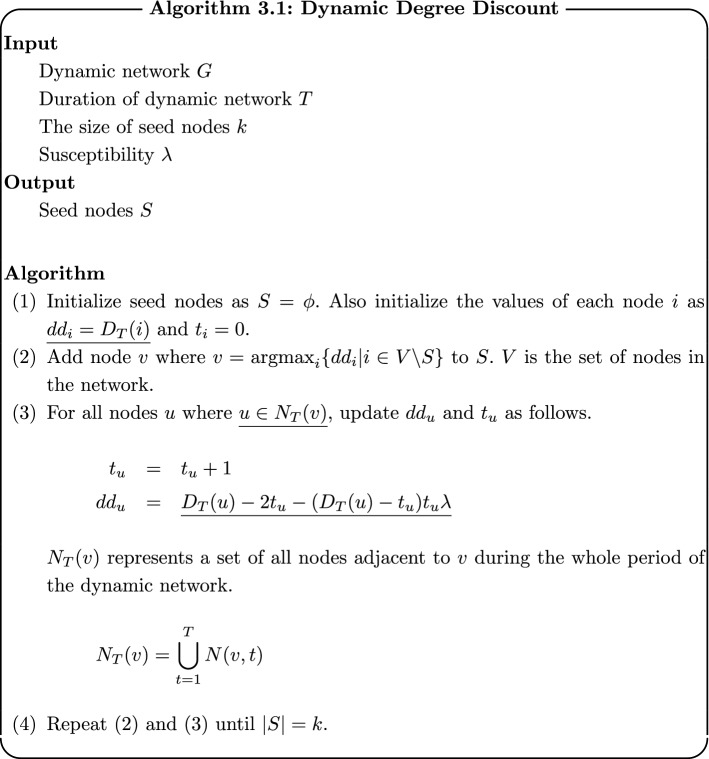


### Dynamic CI

Dynamic CI is an extension of Morone’s method [18] for dynamic networks. Morone’s method focuses on the degree of node *v* and the degrees of nodes with distance *l* from *v*. Dynamic CI defines an index $\mathrm{D\_CI}_l (v)$ in which degree and distance are extended to dynamic networks.

$$\begin{aligned} \mathrm{D\_CI}_l (v) = D_T (v) \sum _{u \in DBall(v, l)} D_T (u) \end{aligned}.$$

The differences between $\mathrm{CI}_l (v)$ and $\mathrm{D\_CI}_l (v)$ are as follows: (1) the definition of degree is changed to that for dynamic networks and (2) $\partial Ball(v,l)$ in $\mathrm{CI}_l (v)$ is changed to *DBall*(*v*, *l*). *DBall*(*v*, *l*) represents nodes where their shortest duration of time (mentioned in “Model of information propagation” section) from node *v* is *l*. *l* is a parameter which takes the value within the range $1 \le l \le T$. In the algorithm of Dynamic CI, $\mathrm{D\_CI}_l (v)$ is computed for each node and top *k* nodes are selected as seed nodes.

### Dynamic RIS

Dynamic RIS is an extension of Borgs’s method [20] and Tang’s method [21] for dynamic networks. 
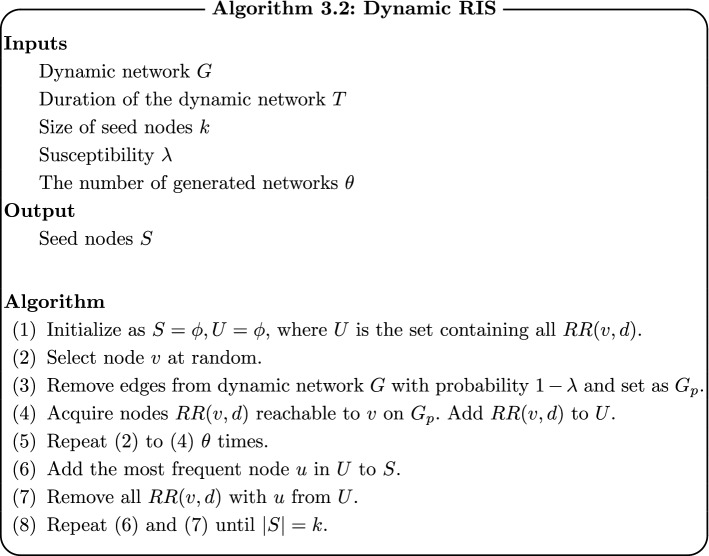


The difference between Borgs’s and Tang’s algorithm and Dynamic RIS is where *RR* in their algorithm is set as *RR*(*v*, *d*) in our algorithm. *RR*(*v*, *d*) is a set of all nodes that are reachable to *v* within the shortest duration of time *d* in all durations of dynamic networks, which is defined as follows:

$$\begin{aligned} RR(v,d) = \bigcup _{t=1}^T RR_t (v,d). \end{aligned}$$

$RR_t (v,d)$ is a set of nodes which are reachable to “node *v* at time *t*” within the shortest period of *d*.

The computational complexities of these methods are as follows.

#### Dynamic Degree Discount

According to the paper of Chen et al. [17], the computational complexity of Degree Discount is $O(k\cdot \text{log} n + m)$, where *k* is the number of seed nodes, *n* is the number of nodes, and *m* is the number of edges, respectively. Dynamic Degree Discount is an extension of Degree Discount. Static degree is replaced with dynamic one ($D_{T}(i)$) and Static neighbors is replaced with dynamic one ($N_{T}(v)$). Computational complexity for dynamic degree and dynamic neighbors is $\frac{T\cdot m}{n}$, where *T* is the total duration of time of given dynamic network. Therefore, the total computational complexity of Dynamic Degree Discount is $O(k\cdot \text{log} n + m + \frac{T\cdot m}{n})$.

#### Dynamic CI

According to the paper of Morone and Makse [18], the computational complexity of CI is $O(n\cdot \text{log} n)$, where *n* is the number of nodes. Dynamic CI is an extension of CI. Static degree is replaced with dynamic one ($D_{T}(i)$), and its computational complexity is $\frac{T\cdot m}{n}$, where *T* is the total duration of time of given dynamic network. Therefore, the total computational complexity of Dynamic CI is $O(n\cdot \text{log} n + \frac{T\cdot m}{n})$.

#### Dynamic RIS

According to the paper of Tang et al. [21], the computational complexity of RIS is $O(k\cdot l^{2}(m + n)log^{2}n/\epsilon ^{3})$ which returns $(1-\frac{1}{e}-\epsilon )$-approximate solution with at least $1-n^{-l}$ probability, where *l* and $\epsilon$ are the constants. Computational complexity of Dynamic RIS heavily depends on the parameters $\theta$ and *d*, which are the number of generated networks and the duration of time for computing *RR*(*v*, *d*), respectively. Therefore, the total computational complexity of Dynamic RIS is $O(\theta \cdot d \cdot k\cdot l^{2}(m + n)log^{2}n/\epsilon ^{3})$.

## Experiments

We perform experiments for comparing the proposed methods with previous ones in order to confirm their effectiveness. Dynamic networks used for the experiments are shown in Table 1. These networks are the same as the ones used in previous research. Average degree in Table 1 shows the average of all nodes in the network, which is $\frac{1}{|V|}\sum _{v \in V} D_T(v)$. Hospital [27] is a network about contacts of patients and medical staffs at hospital with time. Primary School [28, 29] is a network about contacts of students and teachers at school. High School 2013 [30] is a network of contacts of students. The unit of the duration in these three datasets is 20 s. Each dataset is available at SocioPatterns (http://www.sociopatterns.org).

**Table 1:** Dataset for the experiments

	Nodes	Edges	Duration	Ave. deg.
Hospital	75	32,424	9,453	69.3
Primary School	242	125,773	3,100	142.7
High school 2013	327	188,508	7,375	63.0

Methods used in the experiments are previous two methods (Monte-Carlo simulation (MC Greedy) and Osawa) for dynamic network explained in “Influence maximization methods for dynamic networks” section and our proposed methods (Dynamic Degree Discount, Dynamic CI, and Dynamic RIS) in “Proposed methods” section. Given a network as input, each method computes seed nodes *S*. The simulation of influence maximization based on SI model is repeated *R* times with the obtained seed nodes and set the average of the number of nodes in state I as $\sigma (S)$. The values of $\sigma (S)$ are compared in order to evaluate the methods.

Experiments are performed for the following purposes:

1. Comparison of $\sigma (S)$ when the size of seed nodes *k* changes.
2. Comparison of computational time when the size of seed nodes *k* changes.

Parameters in the experiments are set as follows. The number of repetition of the simulations for information propagation is set as $R=50$. The number of repetition of Monte-Carlo simulation in MC Greedy is set as 1000. These two parameters are common in all experiments. The size of seed nodes *k* is set from 0 to 20%. Susceptibility $\lambda$ is set as $\lambda = 0.01$. It is difficult to perform experiments for all the values as parameter *l* in Dynamic CI which takes the value of $1 \le l \le T$. We use the values $l=1, 5, 10, 20$ in the experiments. As the parameters $\theta$ and *d* in Dynamic RIS, $\theta$ is set as $\theta = 1000$ and as for *d*, values $d=0,5,10,20$ are used since it is difficult to perform experiments for all the value as in *l* of Dynamic CI.

CELF [31] is used to speedup the experiments when greedy algorithms are used in MC Greedy and Osawa. CELF is an algorithm used when the greedy algorithm is applied to the problem with inferior modularity, and the solution is the same as in normal greedy algorithm. According to the experiments by Lescovec [31], computational time is 700 times faster than normal greedy algorithm when CELF is used.

**Fig. 4:**
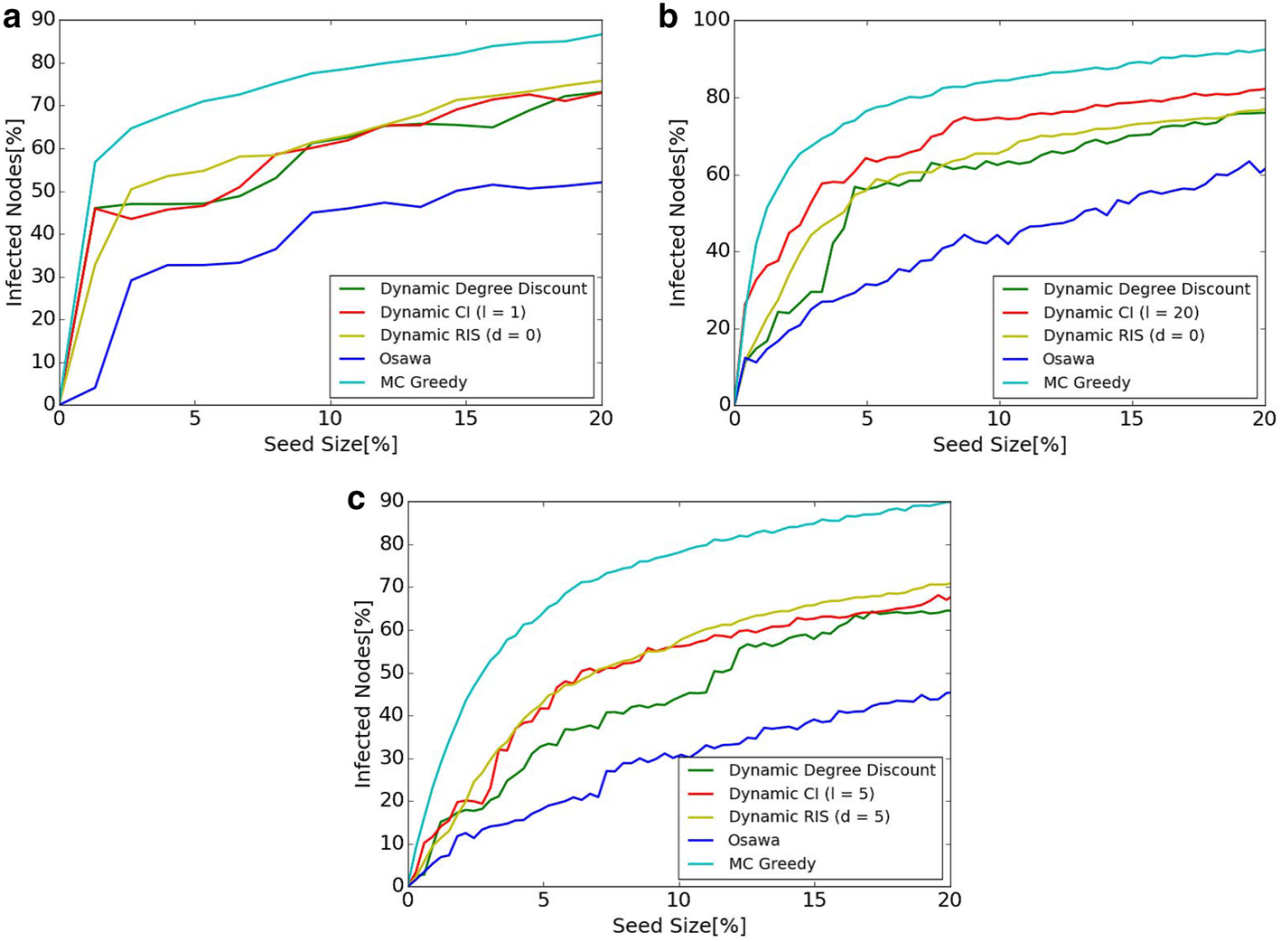
Comparison of $\sigma (S)$ when the size of seed nodes *k* changes. **a** Hospital. **b** Primary School. **c** High School 2013

## Experimental results

### Comparison of $\sigma (S)$ when the size of seed nodes *k* changes

The results of information propagation for each size of seed nodes *k* with fixed susceptible $\lambda = 0.01$ of SI model are shown in Fig. 4. The *x* axis of the Figure shows the percentage of seed nodes, and the *y* axis shows the number of infected nodes. Values of the *x* axis is $\frac{k}{|V|} \times 100$, the percentage of seed nodes to all nodes in the network. Values of the *y* axis is $\frac{\sigma (S)}{|V|} \times 100$, the percentage of $\sigma (S)$ to all nodes in the network. The best values of *l* in Dynamic CI and *d* in Dynamic RIS are used in our experiments. As shown in Fig. 4, MC Greedy achieves the highest diffusion in all datasets. Diffusion of the proposed methods, Dynamic Degree Discount, Dynamic CI, and Dynamic RIS are inferior to MC Greedy, but they are still better than Osawa. The scale of diffusion of Dynamic RIS in High School 2013 achieves 1.5 times as in Osawa.

There is not much difference in the scale of diffusion among each of the three proposed methods. Dynamic RIS achieves the highest in High School 2013, for example, but the difference among proposed methods is small compared with the difference between proposed methods and previous methods (MC Greedy and Osawa).

### Comparison of $\sigma (S)$ when susceptibility $\lambda$ changes

**Fig. 5:**
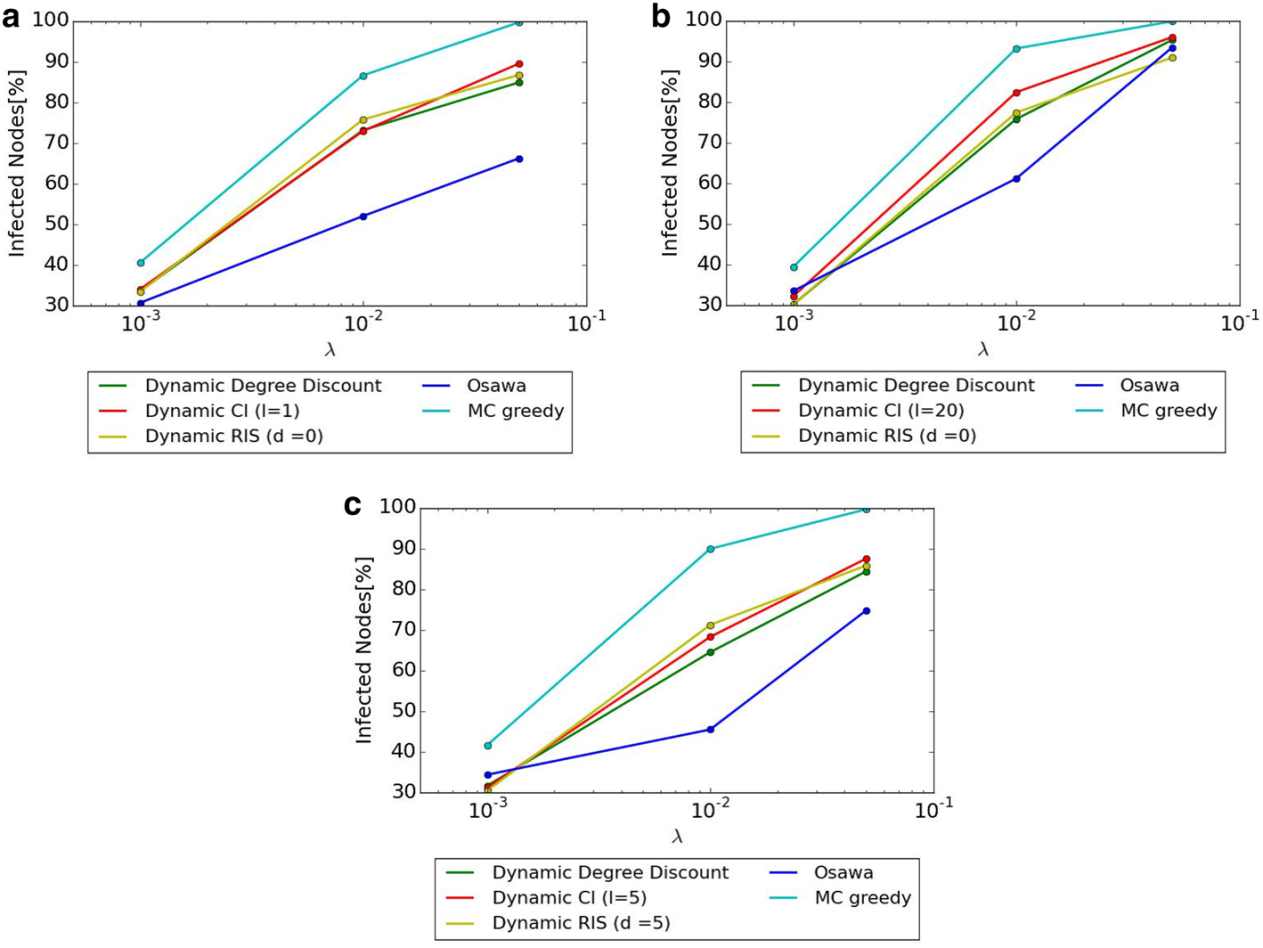
Comparison of $\sigma (S)$ when susceptibility $\lambda$ changes. **a** Hospital. **b** Primary School. **c** High School 2013

Figure 5 shows diffusion when the size of seed nodes is fixed as 20% of all nodes in the networks and susceptibility is changed as $\lambda = 0.001, 0.01, 0.05$. The *x* axis shows the value of $\lambda$, and the *y* axis shows the percentage of diffusion. Parameters *l* and *d* are the same as the ones used in the previous experiments. As shown in Fig. 5, MC Greedy achieves the highest diffusion regardless of the value of $\lambda$. The difference among three proposed methods is small.

As the result of comparison with proposed methods and Osawa, our proposed methods achieve higher scale of diffusion than Osawa in Hospital and High School 2013 when $\lambda = 0.05$. Osawa achieves higher diffusion than Dynamic RIS only in Primary School. When $\lambda = 0.001$, the difference between proposed methods and Osawa is very small compared with the cases of other $\lambda$ values.

### Comparison of computational time when the size of seed nodes *k* changes

**Fig. 6:**
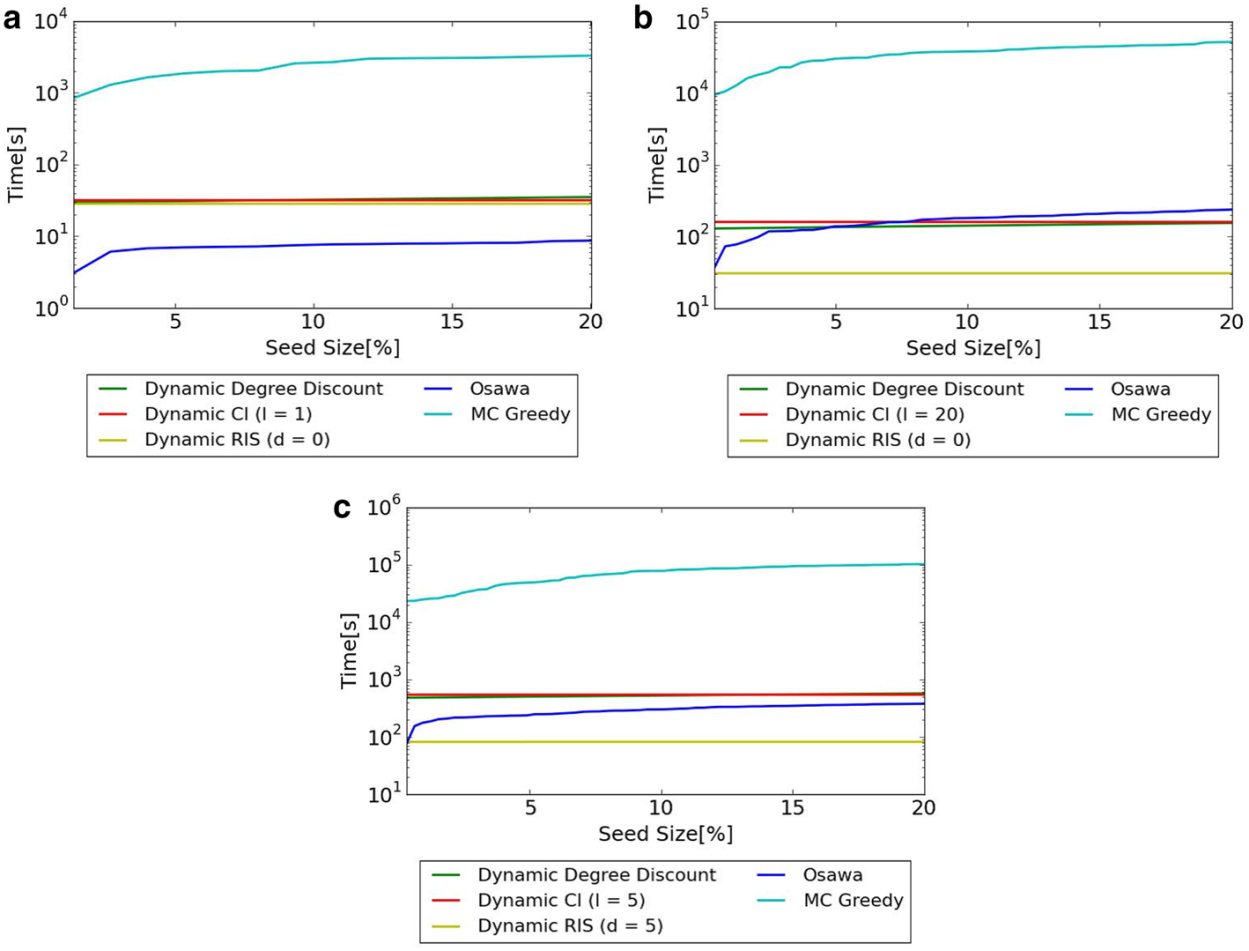
Comparison of computational time when the size of seed nodes *k* changes. **a** Hospital. **b** Primary School. **c** High School 2013

Figure 6 shows the computational time when $\lambda$ is set as $\lambda = 0.01$ and the sizes of seed nodes are changed. A PC of Intel Core i7(3.4 GHz) CPU and 8 GB memory is used for the experiments. *X* axis shows the percentage of seed nodes, and *y* axis shows the computational time (log-scale).

Figure 6 shows that for all datasets, methods other than MC Greedy can compute seed nodes in realistic time. MC Greedy needs several hours to compute seed nodes. This shows that MC Greedy is intractable in realistic time for large-scale networks.

Regarding the comparison among three proposed algorithm, computational time of Dynamic Degree Discount and Dynamic CI is almost the same in all datasets. Dynamic RIS is about the same computational time as the other two proposed methods in Hospital, and is faster in Primary School and High School 2013. Regarding the comparison with proposed methods and Osawa, Dynamic RIS is approximately 7.8 times faster than Osawa except very small network (Hospital).

**Fig. 7:**
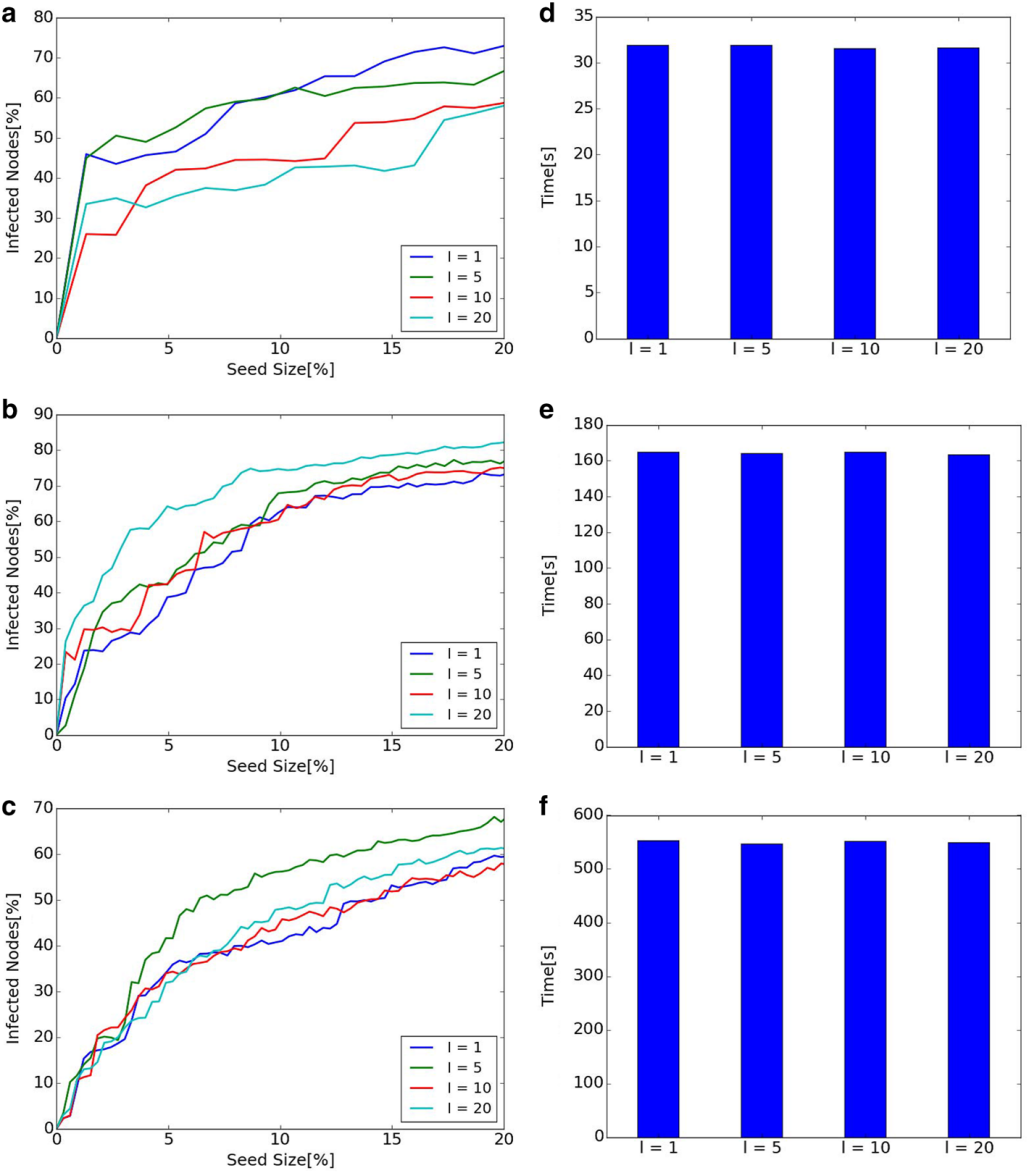
Diffusion and computational time for different *l* in Dynamic CI. **a** Diffusion of Hospital. **b** Diffusion of Primary School. **c** Diffusion of High School 2013. **d** Computational time of Hospital. **e** Computational time of Primary School. **f** Computational time of High School 2013

**Fig. 8:**
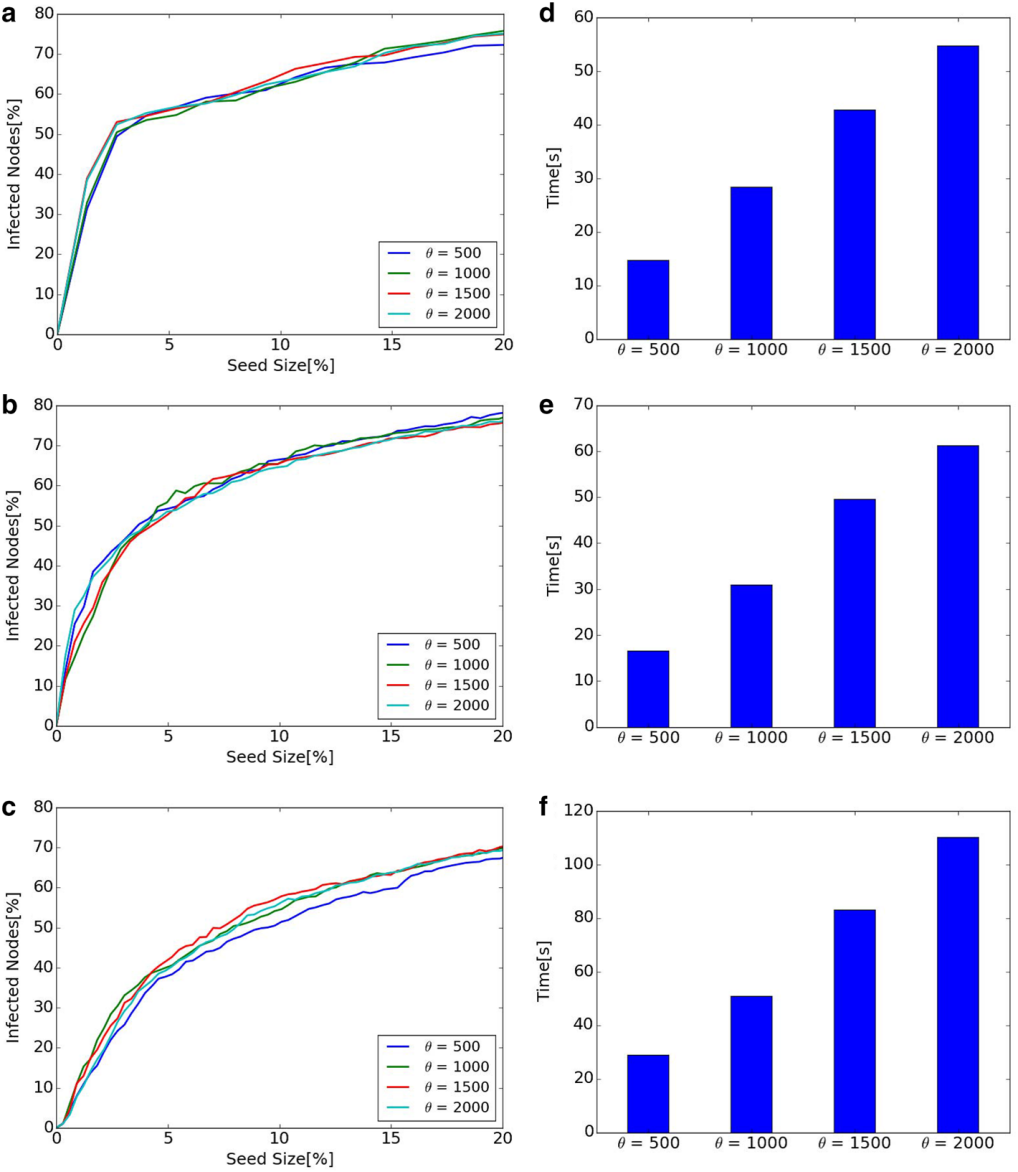
Diffusion and computational time of different $\theta$ in Dynamic RIS. **a** Diffusion of Hospital. **b** Diffusion of Primary School. **c** Diffusion of High School 2013. **d** Computational time of Hospital. **e** Computational time of Primary School. **f** Computational time of High School 2013

**Fig. 9:**
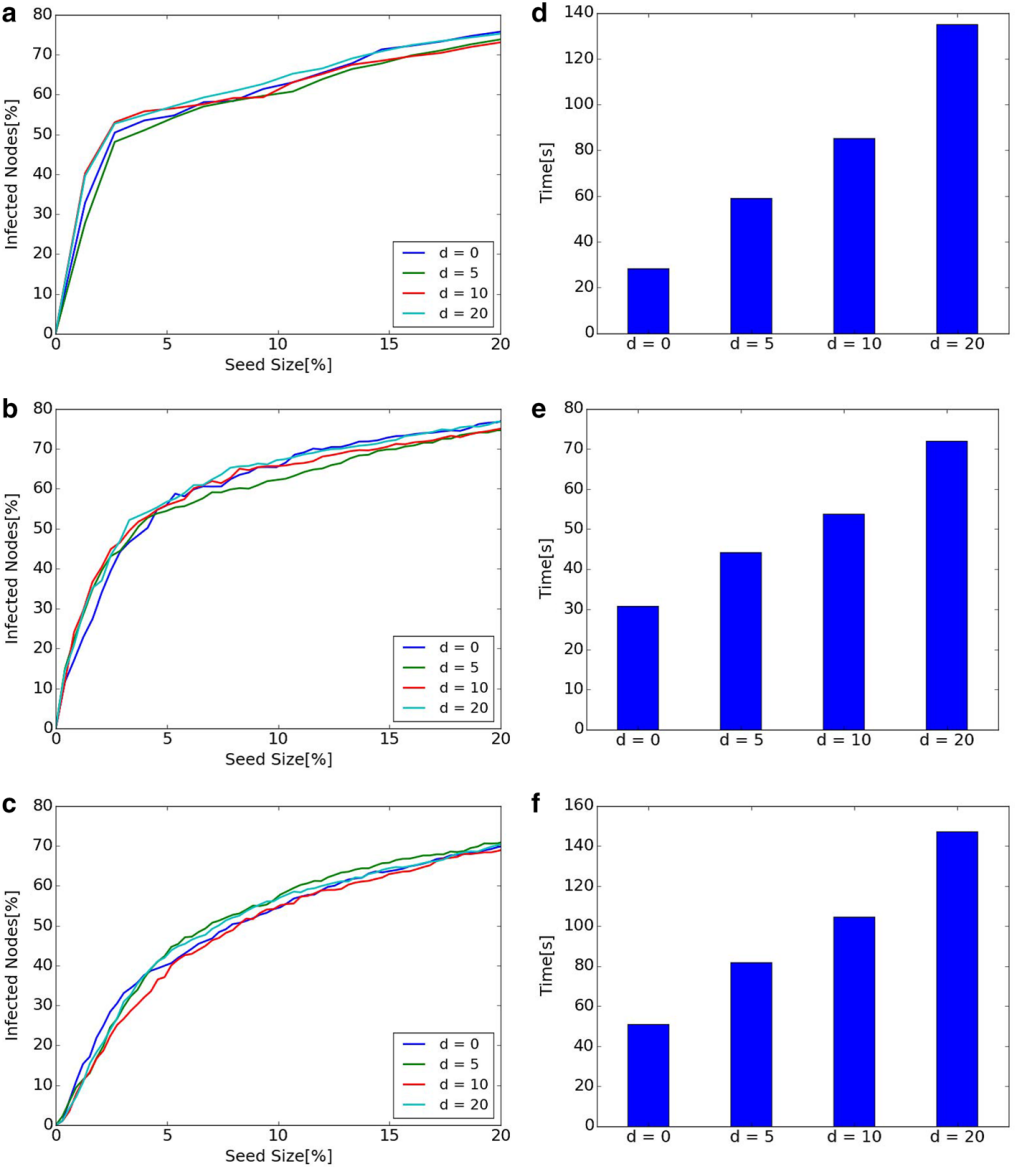
Diffusion and computational time of different *d* in Dynamic RIS. **a** Diffusion of Hospital. **b** Diffusion of Primary School. **c** Diffusion of High School 2013. **d** Computational time of Hospital. **e** Computational time of Primary School. **f** Computational time of High School 2013

### Parameters of Dynamic CI and Dynamic RIS

Diffusions of proposed methods with different parameters are shown in this section. We change parameters *l* of Dynamic CI, and $\theta$ and *d* in Dynamic RIS.

#### Diffusion and computational time of different *l* in Dynamic CI

Diffusion and computational time when *l* in Dynamic CI changes to 1, 5, 10, 20 are shown in Fig. 7. Left line graphs show the size of diffusion when *l* is changed in each network. Right bar graphs show computational time. Left line graphs show that diffusion depends on the value of *l*. Therefore, it is important to find appropriate *l* in Dynamic CI. Since there is no simple correlation between the scale of diffusion and the value of *l* (such as diffusion becomes larger as *l* becomes large), diffusions for various values of *l* should be investigated and compared. Right bar graphs show that there are no big differences of execution time when the value of *l* changes.

#### Diffusion and computational time of different $\theta$ in Dynamic RIS

$\theta$ in Dynamic RIS is a parameter for the number of generated graphs in *RR*(*v*, *d*). Diffusion and computational time when parameter $\theta$ is changed to 500, 1000, 1500, 2000 are shown in Fig. 8. Left line graphs show that diffusion does not change much when $\theta$ changes. However, the scale of diffusion is slightly small when $\theta = 500$ in Hospital and High School 2013. This means that bigger $\theta$ is desirable from the viewpoint of diffusion. On the contrary, right bar graphs show that higher value of $\theta$ results in the increase of computational time. From the viewpoint of computational time, smaller $\theta$ is better. Regarding the value of $\theta$, there is a trade-off between the scale of diffusion and the computational time. It is important to find smaller $\theta$ for shorter computational time, but too small $\theta$ results in small-scale diffusion.

#### Diffusion and computational time of different *d* in Dynamic RIS

*d* in Dynamic RIS is a parameter for the number of time steps for looking back. Figure 9 shows diffusion and executing time when parameter *d* changes to 0, 5, 10, 20. Left line graphs show that there is almost no difference in diffusion when *d* changes, while right bar graphs show that computational time increases as the value of *d* becomes bigger. The scale of diffusion does not change even if the value of *d* becomes bigger in our experiments.

## Discussion

### Analysis focused on expansion of each node

In the experiments when susceptibility changes in “Advantages and disadvantages of each of the proposed methods” section, the difference between the proposed methods and Osawa was small when $\lambda = 0.001$ compared with the experiments with other values of $\lambda$. When $\lambda = 0.05$, Osawa outperforms proposed methods only in Primary School. This section discusses these two points.

**Fig. 10:**
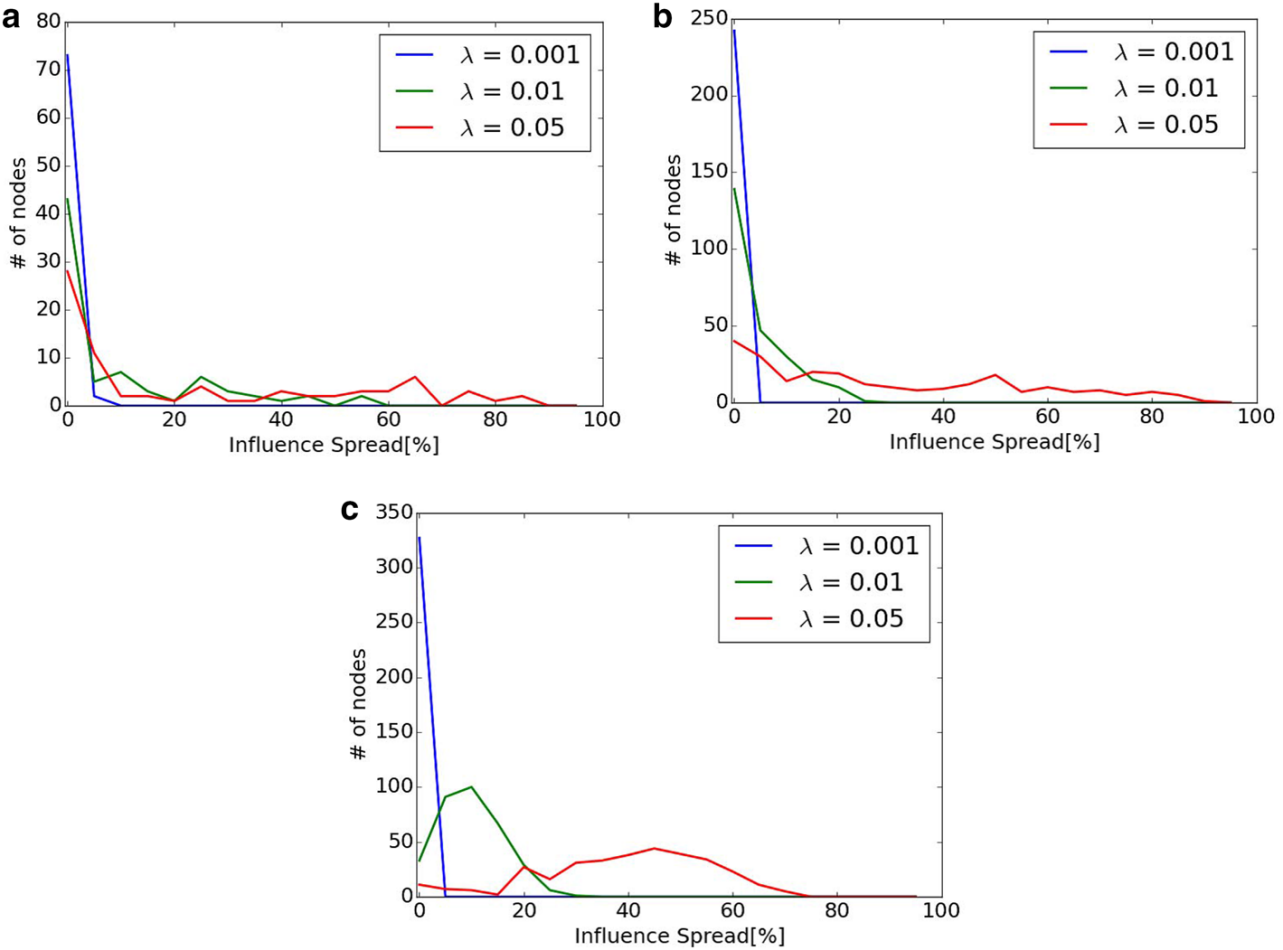
Distribution of diffusion $\sigma (\{ v \} )$ of each node *v*. **a** Hospital. **b** Primary School. **c** High School 2013

Figure 10 shows the distribution of diffusion $\sigma (\{ v \})$ of each node *v* when Monte-Carlo simulation is used. *X* axis shows the percentage of diffusion from node *v* to the whole network ($\sigma (\{ v \})$), and *Y* axis shows the frequency of the nodes with each of the percentage in *X* axis. When $\lambda = 0.001$, almost all nodes are less than 5% of diffusion in all networks. This means that there is no big difference of the diffusion from different seed nodes. This is the reason why the difference between proposed methods and Osawa is small in the experiment in “Advantages and disadvantages of each of the proposed methods” section. On the contrary, there are many nodes with more than 60% of diffusion in Primary School when $\lambda = 0.05$ compared with other two networks. In this case, large-scale diffusion is easy to be achieved even if the most appropriate seed nodes are not selected. This is the reason why Osawa outperforms proposed method in Primary School in “Advantages and disadvantages of each of the proposed methods” section.

### Advantages and disadvantages of each of the proposed methods

Advantages and disadvantages of each of the proposed methods are discussed in this section. An advantage of Dynamic Degree Discount is that it contains no parameter, so there is no need to adjust parameter. Its disadvantage is that it is only for SI model, so the method cannot be used for other models. This is because Dynamic Degree Discount is an extension of Chen’s Degree Discount which is for SI model. There are other information propagation models such as LT model and Triggering models proposed by Kempe et al. Dynamic Degree Discount cannot be applied to such models.

An advantage of Dynamic CI is that it can be applied to many information propagation models in contrast to Dynamic Degree Discount because Dynamic CI uses only degree information when it calculates seed nodes. Its disadvantage is that the ability of diffusion depends on the value of parameter *l* as mentioned in “Diffusion and computational time of different *l* in Dynamic CI” section. It is necessary to search for appropriate values of *l* for Dynamic CI. The parameter *l* takes the value within the range $1< l < T$, so the search takes time in general.

An advantage of Dynamic RIS is that its computational time is short. As shown in the experimental results, its computational time is shorter than other methods in all networks except Hospital. As the method can be applied to large networks due to its short computational time, this is a big advantage. Disadvantage of Dynamic RIS is that it needs to adjust parameters $\theta$ and *d*. As mentioned in the previous section, computational time becomes bigger as the parameter $\theta$ becomes bigger, and the scale of diffusion becomes smaller for too small $\theta$. Therefore, it is necessary to set appropriate value for $\theta$. However, parameter sensitivity of $\theta$ and *d* is not so much compared with the sensitivity of *l* in Dynamic CI.

## Conclusion

We propose three new methods for influence maximization problem in dynamic networks which are the extensions of the methods for static networks. As the result of experiments for comparing with previous methods, MC Greedy and Osawa, our three proposed methods are better than previous methods in the following sense. Although the performance of MC greedy is better than these three methods, it is computationally expensive and intractable for large-scale networks. The computational time of our proposed methods is more than 10 times faster than MC greedy, so they can be computed in realistic time even for large-scale dynamic networks. When compared with Osawa, the performances of these three methods are almost the same as Osawa, but they are approximately 7.8 times faster than Osawa. Based on these facts, the proposed methods are suitable for influence maximization in dynamic networks.

The comparison of Dynamic Degree Discount, Dynamic CI, and Dynamic RIS is as follows. The choice of the methods should be done based on the following pros and cons.

**Dynamic Degree Discount**

- It requires no parameter.
- It is applicable to SI model only.

**Dynamic CI**

- It is applicable to other information propagation models.
- The performance heavily depends on parameter *l*.

**Dynamic RIS**

- It is relatively fast among these three methods.
- It requires two parameters to be adjusted ($\theta$ and *d*).

Finding the strategies of choosing suitable method for given dynamic network is practically important. It is a challenging open question and is left for our future work.

The problem of adjusting the parameters for Dynamic CI and Dynamic RIS is also left for our future work.
